# Strength–Ductility Synergy and Microscopic Mechanism of CNTs-Reinforced Mg-Al Composites Fabricated Through Vacuum Powder Metallurgy Coupled with Hot Extrusion–Rolling

**DOI:** 10.3390/ma19081537

**Published:** 2026-04-12

**Authors:** Shiwei Ma, Guo Li, Ning Zhang, Shaojian Huang, Hao Chen, Guobing Wei, Jinxing Wang

**Affiliations:** 1 National Engineering Research Center for Magnesium Alloys, Chongqing University, Chongqing 400044, China; 2 International Joint Laboratory for Light Alloys (MOE), Chongqing University, Chongqing 400044, China; 3 Department of Materials Science and Engineering, University of Toronto, 184 College Street, Toronto, ON M5S 3E4, Canada

**Keywords:** magnesium matrix composites, CNTs, microstructural evolution, strength–ductility synergy, powder metallurgy, hot extrusion–rolling

## Abstract

The low absolute strength and insufficient room-temperature ductility remain key bottlenecks that restrict the engineering application of magnesium alloys in high-end industrial fields. In the present study, 1 vol.% carbon nanotubes (CNTs)-reinforced Mg-xAl (x = 0, 1, and 1.5 wt.%) composites were synthesized via a powder metallurgy route coupled with hot extrusion–rolling processing to realize a simultaneous improvement in mechanical properties. The hot extrusion–rolling processed 1 vol.% CNTs/Mg-1Al composite exhibits an ultimate tensile strength of 300 MPa and an elongation to failure of 9%, showing an excellent strength–ductility synergy. Microstructural characterization reveals a well-bonded interface between CNTs and the Mg matrix. Deformation incompatibility between CNTs and the magnesium matrix during hot extrusion–rolling induces a high density of dislocations, providing an important strengthening contribution. Moreover, an increased proportion of low-angle grain boundaries and the development of a bimodal texture promote significant grain refinement and effectively activate non-basal slip systems, thereby alleviating plastic deformation constraints. The synergistic effects of interfacial strengthening, dislocation strengthening, grain boundary strengthening, and texture regulation together contribute to the simultaneous improvement of strength and ductility in CNTs-reinforced Mg-Al composites.

## 1. Introduction

As one of the lightest metallic structural materials for industrial engineering applications, magnesium alloys feature a low density of around 1.74 g/cm^3^, which is roughly one-quarter of that of steel. In addition, magnesium alloys have the advantages of high specific strength, favorable electromagnetic shielding performance, and excellent biocompatibility, rendering them promising candidates for aerospace applications, automotive manufacturing, electronic devices, and biomedicine [1,2,3,4]. However, the insufficient strength and restricted ductility of magnesium alloys significantly hinder their widespread application as structural components. Therefore, further improvement of their mechanical properties is required to satisfy practical service demands.

Alloying and plastic deformation are commonly employed approaches to optimize the mechanical performance of metallic materials. For magnesium alloys, the addition of Al can improve tensile strength through solid-solution strengthening of the Mg matrix and can also contribute to grain refinement [5,6,7]. Moreover, subsequent plastic deformation processes, such as hot extrusion and hot rolling, are effective in tailoring the grain structure, thereby improving the mechanical properties of magnesium alloys [8,9,10]. Nevertheless, strength enhancement in magnesium alloys is often accompanied by a reduction in ductility. Consequently, achieving a balanced improvement in both strength and ductility remains a core technical challenge [11,12].

Introducing reinforcing particles into magnesium alloys has been widely verified as a feasible approach to solve this bottleneck. However, micron-scale reinforcements, including ceramic and carbon-based particles, generally improve strength at the expense of ductility [13,14]. In contrast, nanoscale reinforcements, by virtue of their ultrahigh specific strength and microstructural regulation capability, have been proven to boost the comprehensive mechanical properties of composites at relatively low addition levels [13]. Among these reinforcements, CNTs, characterized by unique nanostructures and geometrical features (e.g., ultrahigh aspect ratio and huge specific surface area), low density, and excellent mechanical properties (tensile strength ranging from 11 to 63 GPa and elastic modulus of around 1 TPa), are widely regarded as effective reinforcements for metal matrix composites [6,15]. However, CNTs are prone to agglomeration owing to the strong intertube van der Waals forces and electrostatic interactions, which can hinder composite densification and deteriorate mechanical properties [16,17,18]. Therefore, realizing homogeneous dispersion of CNTs in the Mg matrix is recognized as a vital precondition to fully unlock their reinforcing potential.

The powder metallurgy (PM) process, due to its excellent material mixing uniformity and particular suitability for dispersing nanoscale reinforcements prone to agglomeration, has been verified as one of the most competitive techniques for preparing CNTs-reinforced magnesium matrix composites [15,19]. This process typically involves ball milling to uniformly mix metal powders with reinforcing particles, followed by sintering to produce consolidated materials [19,20]. Present studies [21] have reported that when the CNTs content is ≤1 vol.%, CNTs can achieve relatively homogeneous dispersion via the PM route, leading to improved yield strength and tensile strength with rising CNTs content. In contrast, when the CNTs content exceeds 1 vol.%, CNT agglomeration becomes more pronounced, leading to a deterioration in composite strength. Accordingly, 1 vol.% CNTs were selected in this study to balance dispersion and strengthening efficiency.

To further optimize the mechanical performance of the as-fabricated composites, subsequent deformation processing, such as hot extrusion or hot rolling, is typically required. Gao et al. [22] and Dong et al. [23] fabricated B4Cp/6061Al composites and CNTs-reinforced Ti-6Al-4V matrix composites, respectively, via powder metallurgy, followed by rolling, achieving a synergistic improvement in both strength and ductility. Wang et al. [24] successfully prepared ultrafine-grained AZ31 magnesium alloy through powder metallurgy combined with subsequent hot extrusion and rolling, attaining a maximum superplastic elongation of 227%. In addition, Liao et al. [25] fabricated a high-ductility Mg-8.10Al-0.42Zn-0.51Mn-1.52La-1.10Gd-0.86Y (wt.%) alloy through combined hot extrusion and rolling processes, demonstrating that this process can significantly refine grains and achieve a simultaneous enhancement of strength and ductility. Based on these findings, hot extrusion and rolling were employed as the deformation processing routes in the present study.

Accordingly, 1 vol.% CNTs/Mg-xAl (x = 0, 1.0, and 1.5 wt.%) composites were fabricated via ball milling, vacuum hot-pressing sintering, and subsequent hot extrusion–rolling. The combined addition of Al and CNTs enables a simultaneous enhancement of strength and ductility in the magnesium matrix composites. Notably, the hot-extruded and rolled CNTs/Mg-1.0Al composite exhibited an ultimate tensile strength of 300 MPa, together with an elongation of 9%. In addition, the mechanisms of strength–ductility synergy in CNTs/Mg-xAl composites are systematically discussed.

## 2. Materials and Methods

### 2.1. Material Preparation

**Figure 1:**
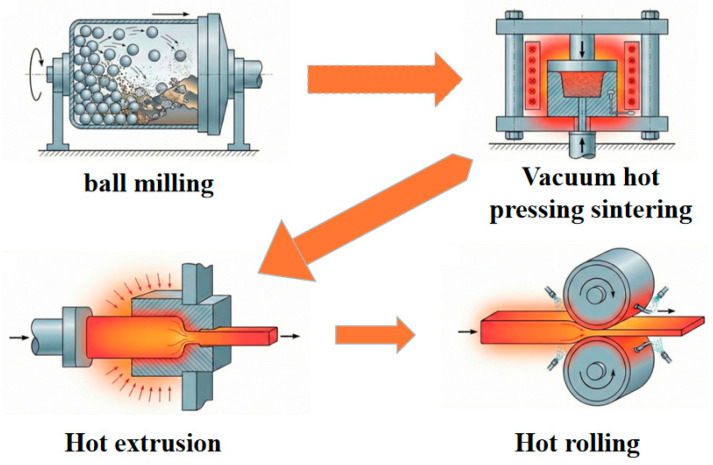
Schematic diagram of the PM and hot extrusion rolling process for preparing CNTs/Mg-xAl composites.

CNTs-reinforced Mg-based composites were synthesized via a powder metallurgy route coupled with a hot extrusion–rolling process. The schematic processing route is shown in Figure 1. Pure magnesium powder (average particle size ~100 μm, purity 99.6 wt.%), pure Al powder (mean particle size ~40 μm, purity 99.8 wt.%), as well as multi-walled CNTs (outer diameter 3–15 μm, length 15–30 μm, purity over 97 wt.%, 1 vol.% with respect to the metal matrix) were placed into a stainless-steel vacuum ball-milling tank (QM-QX4L planetary mill, Changsha Miqi, Changsha, China). Ball milling was conducted at 180 r/min for 3 h under vacuum conditions with a ball-to-powder mass ratio set at 5:1. The milled powder mixtures were consolidated via vacuum hot-pressing at 550 °C under a uniaxial pressure of 30 MPa with a holding time of 2 h to fabricate billets of CNTs/Mg-xAl (x = 0, 1.0, 1.5 wt.%) composites. The core objective of this work is to systematically investigate the influence of Al addition on the microstructure and mechanical properties of CNTs-reinforced Mg composites, with the CNTs/Mg composite (0 wt.% Al addition) set as the control group to isolate the independent effect of Al content. Prior to extrusion, the hot-pressing billets were preheated to 500 °C and held for 180 s under an argon protective atmosphere and then extruded on a 2000 kN hydraulic press (SHP-200-450, Shibayama, Osaka, Japan) with an extrusion ratio of 12:1 and an extrusion rate of 0.5 mm/s. After being air-cooled to room temperature, the specimen was reheated to 300 °C at a heating rate of 10 °C/min, maintained at this temperature for 15 min, and then subjected to a rolling process with a total thickness reduction of 50%.

### 2.2. Characterization of the Composites

**Figure 2:**
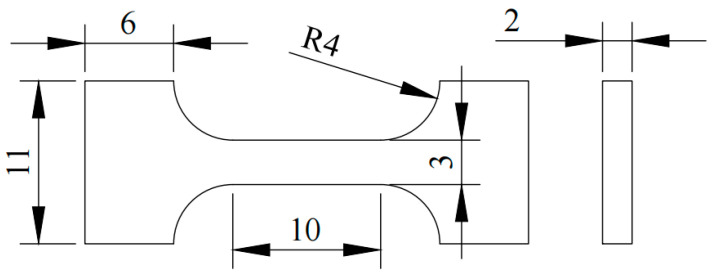
Tensile specimen dimensions (unit: mm).

After mechanical polishing, the hot-extruded and rolled specimens were etched in a picric acid etchant for 10–15 s. Microstructural observations were carried out using optical microscopy (OM, ZEISS Smartzoom5, ZEISS Industrial Quality Solutions, Oberkochen, Germany) and scanning electron microscopy (SEM, JEOL JSM-7800F, JEOL, Tokyo, Japan) equipped with an Oxford Aztec EBSD detector (Oxford Instruments NanoAnalysis, High Wycombe, UK) and an energy-dispersive spectrometer (EDS) (Oxford Instruments NanoAnalysis, High Wycombe, UK). EBSD data were processed using HKL Channel 5 software (Version number: 5.12.74.0). Transmission electron microscopy (TEM, JEOL JEM-2100, JEOL, Tokyo, Japan) was conducted at an accelerating voltage of 200 kV, and TEM foils were prepared using a Gatan 695 cryogenic ion thinning system (Gatan, Inc., Pleasanton, CA, USA). Tensile specimens were machined along the rolling direction (RD), the specimen dimensions are shown in Figure 2, 4 parallel samples per set of process parameters were used for tensile testing to guarantee the repeatability and reliability of the experimental results, and uniaxial tensile tests were performed at a strain rate of 10^−3^ s^−1^.

## 3. Results and Discussion

### 3.1. Microstructural Morphology Analysis

**Figure 3:**
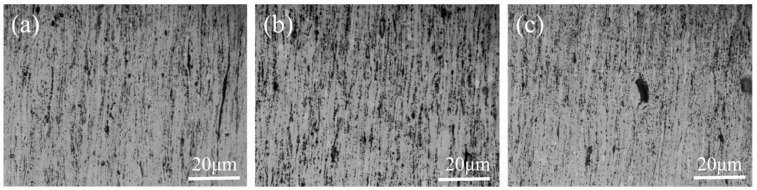
OM images of as-extruded CNTs/Mg-xAl (wt.%) composites. (**a**) x = 0, (**b**) x = 1.0, and (**c**) x = 1.5.

Figure 3 presents OM images of CNTs/Mg-xAl composites along the rolling direction. Discrete black particles aligned along the rolling direction are observed in all composites. After hot extrusion–rolling, these particles are generally well dispersed; however, relatively larger agglomerates are observed in Al-containing composites (Figure 3b,c), which can be attributed to the aggregation of second-phase particles. The agglomeration of the second phase in Figure 3c is more pronounced than that observed in Figure 3b.

**Figure 4:**
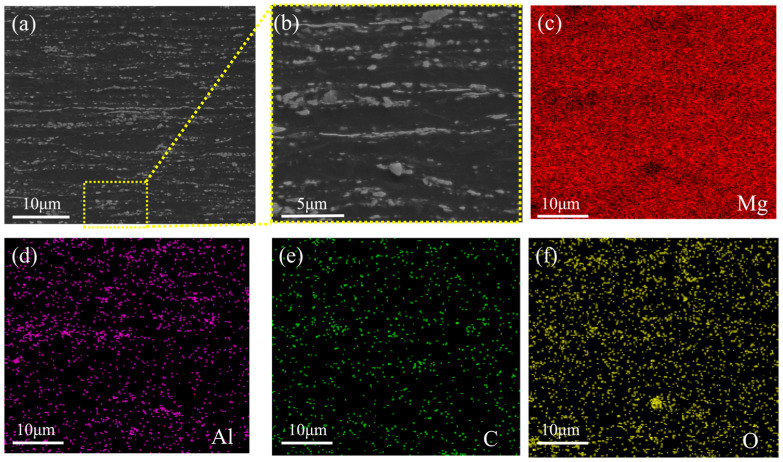
SEM images of CNTs/Mg-1.0Al (wt.%) composites (**a**), (**b**) and surface scanning result of Mg (**c**), Al (**d**), C (**e**), and O (**f**) elements in (**b**).

To further elucidate the microstructural characteristics of the composites, SEM observations coupled with EDS analyses were conducted on the CNTs/Mg-1.0Al composite, as shown in Figure 4. Discrete white particles aligned along the rolling direction are observed in Figure 4a,b, corresponding to the black particles observed in the OM images in Figure 3. The EDS maps (Figure 4c–f) reveal pronounced enrichment of Al and O within these particles. According to the Mg-Al binary equilibrium phase diagram and the high chemical reactivity of magnesium, these particles are inferred to consist primarily of Mg_17_Al_12_ intermetallic compounds and MgO phases. The MgO phase is likely formed through reactions between Mg and trace oxygen during the sintering process. Previous studies have indicated that MgO may serve as an interfacial transition layer, which is beneficial for interfacial adhesion between CNTs and the Mg matrix by facilitating load transfer and regulating interfacial interactions [26,27]. In addition, localized carbon enrichment is detected at the interfaces between Mg-rich and Al-rich regions (Figure 4e). This observation suggests that CNTs are preferentially distributed at these interfaces and may act as preferential heterogeneous nucleation sites, thus facilitating the nucleation and growth of the Mg_17_Al_12_ phase, in good agreement with previous reports [28].

**Figure 5:**
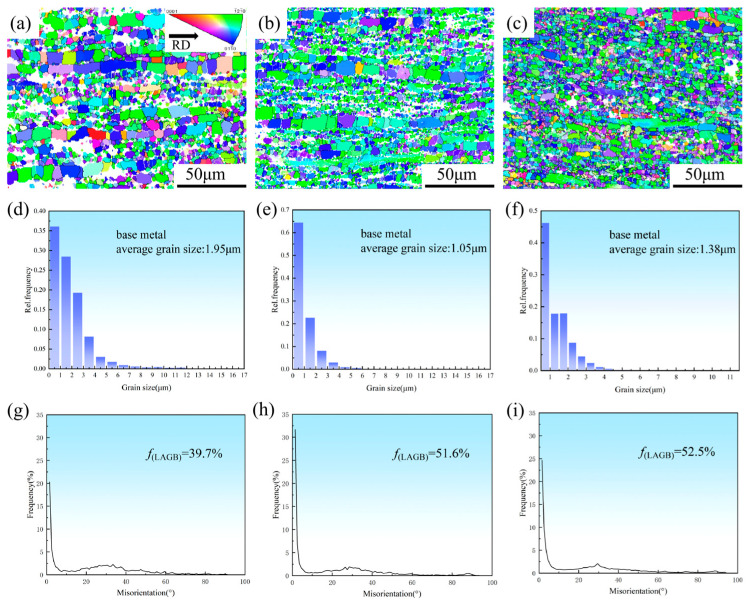
IPF maps, corresponding grain size distribution maps, and misorientation angle distributions of CNTs/Mg-xAl (wt.%) composites. (**a**,**d**,**g**) x = 0, (**b**,**e**,**h**) x = 1.0, and (**c**,**f**,**i**) x = 1.5.

Figure 5 presents inverse pole figures (IPF) maps, grain size distribution profiles, and misorientation angle distribution curves of the extruded and hot-rolled CNTs/Mg-xAl composites acquired via EBSD characterization. As shown in Figure 5a–c, the magnesium matrix is composed of two types of grains, fine dynamically recrystallized (DRX) grains and coarse unrecrystallized grains, after the combined extrusion and rolling process. The grain size distribution diagrams (Figure 5d–f) indicate that the average grain sizes are 1.95 μm for CNTs/Mg, 1.05 μm for CNTs/Mg-1.0Al, and 1.38 μm for CNTs/Mg-1.5Al. The refined grain sizes across all three composites are likely related to the pinning effect of CNTs and fine second-phase particles [29]. Furthermore, the grain boundary misorientation distribution diagrams (Figure 5g–i) reveal a high fraction of low-angle grain boundaries (LAGBs) in all samples. Previous studies have suggested [30,31,32,33] that LAGBs can serve as preferential nucleation sites for secondary phase particles, verifying the identification of the white particles in Figure 4 as Mg_17_Al_12_. In addition, LAGBs may impede dislocation motion, locally increase dislocation density, and provide a driving force for second-phase precipitation. These effects can synergize with grain refinement and precipitation strengthening to improve the comprehensive mechanical properties of the composites.

**Figure 6:**
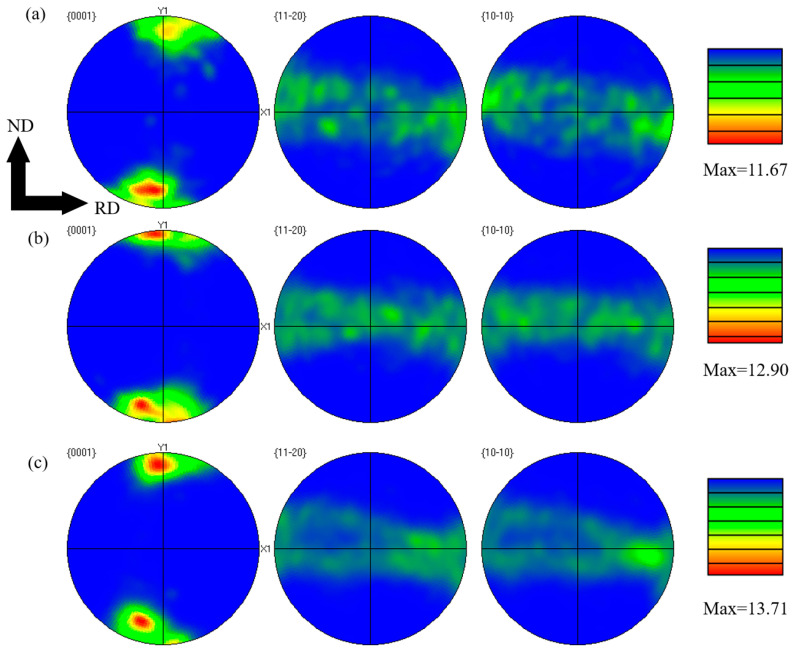
The pole figures with maximum intensity of CNTs/Mg-xAl (wt.%) composites. (**a**) x = 0, (**b**) x = 1.0, and (**c**) x = 1.5.

Figure 6 presents the maximum intensity pole figures of CNTs/Mg-xAl composites. With increasing Al content, the maximum texture intensity of the composites increases from 11.67 (mud) for the CNTs/Mg composite to 13.71 (mud) for the CNTs/Mg-1.5Al composite. The (0001) pole figure exhibits two distinct regions of intensity concentration, indicating the formation of a basal bimodal texture. Such a bimodal basal texture is known to effectively reduce mechanical anisotropy and improve formability. In addition, it can contribute to enhanced mechanical properties by promoting grain refinement, as reported in previous studies [34].

**Figure 7:**
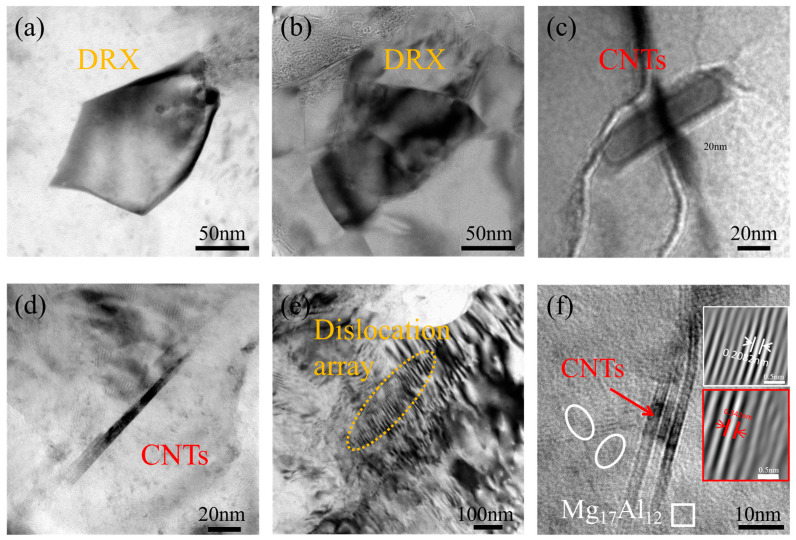
(**a**–**f**) Bright field TEM image of CNTs/Mg-1.0Al (wt.%) composites.

The microstructure of CNTs/Mg-xAl composites was further characterized by TEM, as shown in Figure 7. Equiaxed dynamically recrystallized grains with sizes of 0.1–0.3 μm were observed in the composites after extrusion and hot rolling (Figure 7a,b), indicating the formation of fine-grained structures, consistent with the EBSD results. In addition, numerous dislocations were distributed around these grains. Figure 7c,d present the morphology and distribution of CNTs in the composites, revealing that they are relatively uniformly dispersed in the matrix, with no observable voids, cracks, or other defects at the interface, which is beneficial for enhancing the mechanical properties [35]. Figure 7e shows extensive dislocation arrays formed by dislocation accumulation, indicating significant dislocation activity during plastic deformation and suggesting that dislocation strengthening contributes to improved mechanical properties. Around the CNTs, a large number of nanograins were observed (Figure 7f). Fast Fourier transform (FFT) and inverse FFT (IFFT) analyses confirmed that these nanograins correspond to the Mg_17_Al_12_ phase, further demonstrating that CNTs promote heterogeneous nucleation of the Mg_17_Al_12_ phase [35].

### 3.2. Mechanical Properties

**Figure 8:**
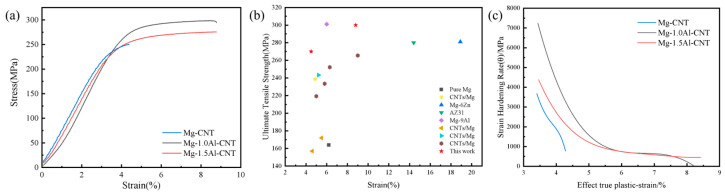
(**a**) The tensile engineering stress–strain curves of extruded CNTs/Mg-xAl composites; (**b**) distribution map of UST and elongation of investigated composites and other kinds of Mg matrix composites reported in the last 5 years [28,35,36,37,38,39,40,41,42], and (**c**) strain hardening curve of CNTs/Mg-xAl composites.

Figure 8a shows the tensile engineering stress–strain curves of CNTs/Mg-xAl composites with varying Al contents. The test results confirm that the co-introduction of Al and CNTs significantly enhances both the mechanical strength and plasticity of the composites. Specifically, the monolithic CNTs/Mg composite exhibits an ultimate tensile strength of 250 MPa and an elongation of 4.4%; the CNTs/Mg-1.0Al (wt.%) composite achieves an ultimate tensile strength of 300 MPa and an elongation of 9%; and the CNTs/Mg-1.5Al (wt.%) composite delivers an ultimate tensile strength of 275 MPa with an elongation of 9%. As the Al addition level rises from 0 to 1.0 wt.%, the yield strength and ultimate tensile strength of the composites increase steadily, while elongation is pronounced improvement, indicating a synergistic enhancement of strength and ductility. However, as the Al content is further raised to 1.5 wt.%, the strength exhibits a slight decline, while the ductility remains largely unchanged. This behavior can be ascribed to the refined grain microstructure obtained in the CNTs/Mg-1.0Al (wt.%) composite (Figure 5e,f), in which grain refinement strengthening is more effective, leading to superior mechanical performance. Figure 8b compares the ultimate tensile strength and elongation to failure of Mg matrix composites fabricated by different processing routes reported over the past five years. It can be clearly seen that the CNTs/Mg-1.0Al (wt.%) composite developed in this study achieves a tensile strength comparable to that of high-alloy magnesium alloys, while exhibiting superior overall mechanical properties compared with pure magnesium and previously reported CNTs/Mg composites. The work hardening rate (θ = dσ/dε)—true plastic strain curves were calculated based on the tensile engineering stress–strain curves, as shown in Figure 8c. The results show that the work hardening behavior of the composites is significantly affected by Al addition: the Al-containing CNTs/Mg-xAl composites exhibit a significantly higher initial work hardening rate than the Al-free CNTs/Mg composite and maintain a slower decline in work hardening rate during the whole plastic deformation process. In the late stage of plastic deformation, the CNTs/Mg-1.0Al and CNTs/Mg-1.5Al (wt.%) composites can still maintain a high work hardening rate, while the work hardening rate of the CNTs/Mg composite drops rapidly and approaches zero at a lower true strain.

### 3.3. Strength–Ductility Synergy Mechanism of CNTs/Mg-xAl Composites

Under typical conditions, an increase in material strength is generally associated with a noticeable reduction in ductility [41]. In contrast, the CNTs/Mg-1.0Al (wt.%) and CNTs/Mg-1.5Al (wt.%) composites exhibit a simultaneous enhancement in both strength and ductility. This behavior can be attributed to the synergistic effects of Al and CNTs [35,43]. Based on the microstructure characterization results and classic metal strengthening theory, the primary and secondary order of the strengthening mechanisms of the composites is clarified as follows: grain refinement strengthening is the primary strengthening source, followed by dislocation strengthening.

The addition of Al and CNTs to the Mg matrix exerts a synergistic effect, leading to significant grain refinement and thereby enhancing strength via grain refinement strengthening [44]. The β-Mg_17_Al_12_ second-phase particles, promoted by both Al and CNTs, act as heterogeneous nucleation sites for DRX, substantially increasing the nucleation rate. Moreover, dissolved Al atoms form “solute atom atmospheres” within the Mg matrix, which impede grain boundary migration, suppress the growth of newly formed DRX grains, and ultimately result in fine and uniform recrystallized grains. As illustrated in Figure 5, the grain dimensions of the two Al-containing specimens are remarkably decreased. According to the classic Hall–Petch relationship [45]:

(1) $$\sigma_{y}=\sigma_{0}+k/\sqrt{d}$$

where σ_y_ is the yield stress, σ_0_ is the lattice friction stress required for dislocation movement inside the grains, d is the mean grain size, and k is the Hall–Petch constant, which is a key material parameter characterizing the strengthening efficiency of grain boundaries. According to the Hall–Petch relationship, smaller grain sizes increase the hindrance of grain boundaries to dislocation motion, resulting in significant improvements in both yield and tensile strengths. Consistent with this theoretical trend, the CNTs/Mg-1.0Al (wt.%) composite has a smaller average grain size than the CNTs/Mg-1.5Al (wt.%) composite, which accounts for its higher tensile strength. Moreover, grain refinement can also enhance ductility. As shown in Figure 5g–i, the fraction of LAGBs is significantly elevated in the Al-containing composites. Previous studies have demonstrated that [29,30,31,32,33] LAGBs can synergize with nanoscale reinforcements, such as CNTs, to suppress the growth of dynamically recrystallized grains through the Zener pinning effect [29], thereby promoting grain refinement. This observation is consistent with the microstructural results presented in Figure 7a,b. In addition, LAGBs can improve the strain-hardening rate by suppressing dislocation annihilation and enhancing dislocation–grain boundary interactions, contributing to higher strength. The high strain energy associated with LAGBs can also facilitate short-range diffusion of Al atoms, providing atomic flux for secondary phase precipitation and promoting the formation of the Mg_17_Al_12_ phase [30]. Consequently, LAGBs can synergize with grain refinement strengthening and precipitation strengthening and achieve strengthening by hindering dislocation slip [31,32,33]. Furthermore, as transitional interfaces between coarse and ultrafine grains, LAGBs can mitigate heterogeneous deformation by accumulating geometrically necessary dislocations (GNDs) and, in conjunction with nanoscale reinforcements such as CNTs, promote dislocation entanglement and the triggering of extra slip systems, thereby improving the overall mechanical properties [30].

**Figure 9:**
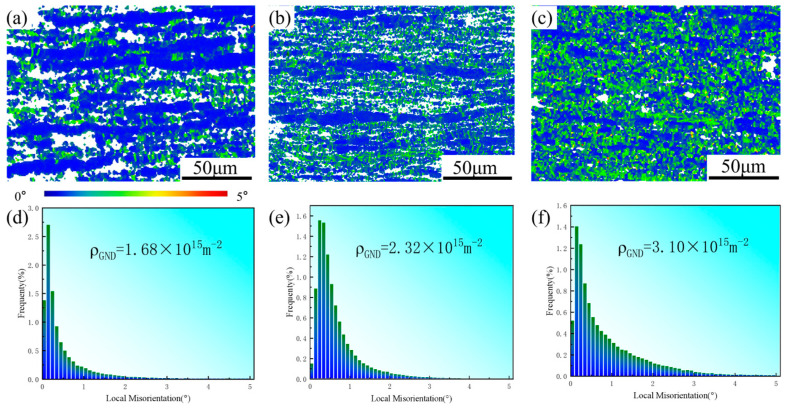
(**a**–**c**) Local misorientation map and (**d**–**f**) corresponding local misorientation angle distribution map of composites. (**a**,**d**) CNTs/Mg composites, (**b**,**e**) CNTs/Mg-1.0Al (wt.%) composites, and (**c**,**f**) CNTs/Mg-1.5Al (wt.%) composites.

The GND density was quantitatively calculated, and the results confirm that the introduction of Al notably raises the GND density. The calculation of GND density was performed using the following formula [43]:

(2) $$\rho_{GNDs}=\frac{2\Delta \theta }{\mu b}$$

where Δθ is the average KAM value; μ is the step size used in the EBSD measurement; and b is the Burgers vector. The calculated results indicate that GND density increases with the Al content: 1.68 × 10^15^ m^−2^ for CNTs/Mg, 2.32 × 10^15^ m^−2^ for CNTs/Mg-1.0Al, and 3.10 × 10^15^ m^−2^ for CNTs/Mg-1.5Al (Figure 9). As discussed above, the addition of Al significantly increases the proportion of LAGBs, which impede dislocation slip and lead to dislocation accumulation and entanglement (Figure 7e). This localized dislocation clustering generates strain gradients, resulting in regions with GND densities substantially higher than the surrounding matrix [30], consistent with the observations in Figure 9a–c. Nevertheless, the CNTs/Mg composite also exhibits a relatively high GND density, attributable to the uniformly dispersed CNTs acting as “dislocation pinning points” that hinder dislocation motion and multiplication. As dislocations encounter the surface of CNTs, they must bypass or shear the nanotubes, further increasing the resistance to dislocation movement. Consequently, CNTs also contribute to dislocation hindrance [46,47,48]. High-density GNDs enhance the work hardening rate of the material, thereby improving the strength of CNTs/Mg-xAl composites [49]. The difference in work hardening behavior directly corresponds to the difference in the GND density of each composite. According to the classic dislocation plasticity theory [49], the work hardening effect of metal materials originates from the continuous proliferation of dislocations, as well as the interaction and entanglement between dislocations, which hinders the movement of dislocations and improves the deformation resistance of the material. GNDs, as the core component of total dislocation density in heterogeneous CNTs/Mg-xAl composites, are the main source of the work hardening effect. The EBSD quantitative results show that the GND density of the composites increases significantly with the increase in Al content, which is completely positively correlated with the work hardening rate level of each composite. A higher and more stable work hardening rate can effectively delay the necking instability during tensile deformation (the necking criterion satisfies θ = σ, where θ is the work hardening rate and σ is the true stress). The high-density GNDs in the Al-containing composites maintain a high work hardening rate during the whole deformation process, which extends the uniform deformation stage before necking, thus achieving a significant improvement in total elongation to failure.

The contribution of dislocation strengthening was analyzed based on the classic Taylor interaction stress model, which is the core formulation of dislocation density-based continuum plasticity theory [50]. The model describes the linear relationship between the flow stress increment caused by dislocation interactions and the square root of dislocation density, with the formula expressed as [50]:

(3) $$\tau_{fl,s}=\alpha \mu b\sqrt{\rho }$$

where τ_fl,s_ is the shear flow stress increment contributed by dislocation strengthening; α is a dimensionless constant related to dislocation interaction types; μ is the shear modulus of pure magnesium; b is the Burgers vector of <a> dislocation in magnesium with HCP crystal structure; and ρ is the total dislocation density. According to the classic Taylor interaction stress model, when the intrinsic material parameters remain unchanged for the same material system, the flow stress increment contributed by dislocation strengthening exhibits a linear positive correlation with the square root of the total dislocation density. As the core component of total dislocation density in heterogeneous composites, the relative change in GND density can directly reflect the evolution trend of total dislocation density. The GND density of all Al-containing composites is significantly higher than that of the Al-free CNTs/Mg control composite; specifically, the GND density increases from 1.68 × 10^15^ m^−2^ (CNTs/Mg composite) to 2.32 × 10^15^ m^−2^ (CNTs/Mg-1.0Al (wt.%) composite). The higher GND density directly brings a greater contribution of dislocation strengthening, which is one of the core reasons for the significantly improved ultimate tensile strength of the Al-containing composites.

Figure 6 shows that all composites exhibit a bimodal texture, which can effectively reduce anisotropy, enhance formability, and optimize mechanical properties through the combined effects of grain refinement and slip/twinning synergy [34,51,52,53]. The presence of two distinct basal orientations in the texture enables the triggering of multiple slip systems (basal, prismatic, and pyramidal <a>), thereby mitigating anisotropy. As reported by Yuan et al. [34], the mechanical performance of these materials can be simultaneously improved through texture strengthening and dislocation strengthening. Specifically, bimodal texture can increase yield strength by reducing the Schmid factor for basal slip, while simultaneously promoting the initiation of extra slip systems. The restricted plasticity of magnesium alloys with a hexagonal close-packed (HCP) structure is mainly ascribed to the limited quantity of readily activated slip systems, poor deformation compatibility, and susceptibility to crack initiation [54]. Consequently, the formation of a bimodal texture also contributes to improved ductility in CNTs/Mg-xAl composites.

**Figure 10:**
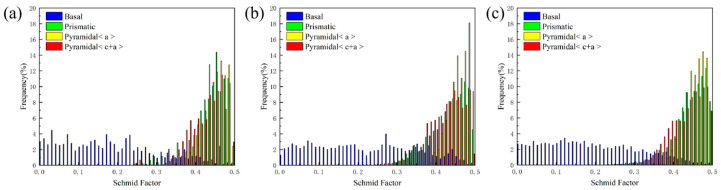
Schmid factor of the various slips in extruded CNTs/Mg-xAl (wt.%) composites. (**a**) x = 0, (**b**) x = 1.0, and (**c**) x = 1.5.

The Schmid factors (SFs) corresponding to various slip systems were characterized via EBSD, and the results are presented in Figure 10. In all composites, the SFs for basal slip are comparatively low, whereas those for prismatic, pyramidal <a>, and pyramidal <c+a> slip systems are elevated. This indicates that, although basal slip requires higher applied stress for activation, other slip systems can be more readily engaged, thereby enhancing the ductility of the composites. Moreover, compared with the CNTs/Mg composite, the SFs for pyramidal <a> slip in the CNTs/Mg-1.0Al (wt.%) and CNTs/Mg-1.5Al (wt.%) composites are increased, particularly for the pyramidal <a> system, suggesting that Al addition promotes the activation of this slip system [55,56,57,58].

Published studies have also confirmed that CNTs promote the initiation of non-basal slip systems [59,60]. For example, Goh et al. [60] reported that in an Mg-1.3CNTs composite, the basal plane is tilted by around 20° along the ND direction, lowering the resolved shear stress required for basal slip and promoting the activation of non-basal slip systems. A similar tilting effect is observed in the CNTs/Mg-xAl composites prepared in this study (Figure 6). Based on critical resolved shear stress (CRSS) data, prismatic <a> and pyramidal <c + a> slip systems at room temperature require approximately 20–25 MPa and 40 MPa, respectively [46]. While basal slip is harder to initiate owing to the low resolved shear stress, dislocation motion can still occur under sufficient stress [60]. CNTs hinder dislocation accumulation, promoting local stress concentrations (Figure 7e) that serve as sources for slip system transformation, thus promoting cross-slip behavior and the initiation of non-basal slip systems [39,60]. Heterogeneous deformation-induced (HDI) hardening also exerts a critical effect on the simultaneous improvement of strength and plasticity in heterogeneous structural materials [61]. The origin of HDI hardening is widely ascribed to the generation of GNDs [61,62]. Our calculations show that a high density of GNDs (ρ_GNDs_ ≈ 2.32 × 10^15^ m^−2^) forms at the interface between CNTs and the magnesium matrix during deformation. These GNDs are long-term pinned by the CNTs, generating internal stress and contributing to hardening, which manifests as improved strength [63]. During plastic deformation, CNTs typically experience greater strain than the surrounding Mg matrix [63]. In this process, GNDs accommodate the strain gradient at the CNTs/Mg matrix interface, guaranteeing the continuity of plastic deformation and thereby helping to preserve the ductility of the material [17,61,62,63].

## 4. Conclusions

In this study, CNTs/Mg-xAl (x = 0, 1.0, 1.5 wt.%) composites with excellent interfacial bonding and uniform CNTs dispersion were successfully fabricated via a “ball milling–vacuum hot-pressing sintering–hot extrusion–rolling” process. This method effectively addresses CNT agglomeration, optimizes interfacial bonding, and enables the synergistic enhancement of both strength and toughness. Among the composites, CNTs/Mg-1Al (wt.%) exhibits a tensile strength of 300 MPa and an elongation of 9%. This outstanding performance is primarily attributed to the inhibition of dynamically recrystallized grain growth by Al, combined with the pinning effect of LAGBs and CNTs, which promotes grain refinement strengthening. In addition, LAGBs hinder dislocation motion, leading to dislocation accumulation and entanglement, thereby enhancing strength through dislocation strengthening. Furthermore, CNTs, LAGBs, and the bimodal texture facilitate the activation of multiple slip systems and improve deformation coordination by increasing the SF of non-basal slip systems. Collectively, these effects result in the simultaneous enhancement of strength and ductility in the composites. The preparation route adopted in this work is an innovative process integration based on the existing magnesium alloy industrial production platform, which can be realized by adapting the existing production lines with low investment. The 1 vol.% CNTs addition level optimized in this study balances the reinforcing effect and processability and will not bring insurmountable obstacles to large-scale production. Economically, the production cost of the composite only has a slight increase compared with commercial magnesium alloys, while its significant lightweight benefits can be converted into considerable indirect economic value in terminal applications. This work provides a feasible technical route for the industrial preparation of high-performance magnesium matrix composites with excellent strength–ductility synergy.

## Data Availability

The original contributions presented in this study are included in the article. Further inquiries can be directed to the corresponding author.
